# Beetroot Powder as Natural Colorant in Fresh Pork Sausages: Impacts on Consumer Liking, Emotional Responses, and Identification of Purchasing Drivers

**DOI:** 10.3390/foods14213715

**Published:** 2025-10-30

**Authors:** Rafaela Willig, Karla Joseane Perez, Lilian Raquel Hickert, Elson Rogerio Tavares Filho, Adriano Gomes da Cruz, Voltaire Sant’Anna

**Affiliations:** 1 Life and Environmental Area, State University of Rio Grande do Sul, Porto Alegre, Encantado Campus, Encantado 96900-000, Brazil; rafaela-willig@uergs.edu.br (R.W.); karla-perez@uergs.edu.br (K.J.P.); lilian-hickert@uergs.edu.br (L.R.H.); 2 Department of Food Technology, Federal Fluminense University, Rio de Janeiro 24230-340, Brazil; elsontavares@live.com; 3 Food Department, Education, Science and Technology Institute from Rio de Janeiro, Rio de Janeiro 20270-021, Brazil; adriano.cruz@ifrj.edu.br

**Keywords:** *Beta vulgaris*, natural dyes, sentence completion, free nitrite/nitrate

## Abstract

The objective of this study was to evaluate the effects of different concentrations of beetroot powder in nitrite-reduced fresh sausages on consumers’ (n = 91) liking, purchase intention, and emotional responses. BP was incorporated into fresh sausage formulations at levels of 3.4 g/kg, 6.8 g/kg, and 10.2 g/kg, alongside a control group without BP. Overall liking was significantly higher for fresh sausages containing BP, regardless of concentration, with color and flavor liking also enhanced by BP addition. Consumer purchasing drivers were identified through the sentence completion method, revealing health benefits and naturalness as the main motivators for purchase. Participants classified as health-oriented showed a greater liking for the samples compared with other consumers, highlighting the impact of health-related factors on purchase intention and emotional responses. In general, health-oriented consumers presented a lower frequency of citations of positive emotions. On average, 63% of participants indicated a willingness to pay approximately 40% more per kilogram for sausages formulated with BP instead of conventional curing salts. In conclusion, BP proved to be an interesting alternative for low/reduced-nitrite/nitrate fresh sausages that appeal to health-conscious consumers.

## 1. Introduction

Beetroot (*Beta vulgaris*) powder is obtained by milling or grinding dehydrated beets and has recently gained attention due to its content of dietary fiber, folic acid, vitamin C, manganese, potassium, zinc, polyphenolics, betalains, and nitrates, among other components [1,2]. Thus, beetroot powder (BP) is a promising functional food ingredient, recognized for its antioxidant, antimicrobial, anticancer, and antiglycation properties [3,4,5]. In this context, the market was valued at approximately USD 456 million worldwide in 2022 and is expected to grow at an annual rate of 5.7% through to 2032 [6].

In the current scenario, BP has demonstrated significant industrial potential in the food and pharmaceutical sectors due to its vibrant red-purple color, serving as a natural alternative to chemical dyes commonly used in large-scale production [2]. The natural color of BP is primarily attributed to the presence of betalains. Betacyanins and betaxanthins, the two main components of betalains, impart attractive red-violet and yellow hues to foods, respectively [2,3,4]. BP has been studied as a natural biopreservative and coloring agent in cured sausages [5], pastırma [6], and milk-based products [7], among others [2,8]. In cured meats, beetroot has emerged as an important alternative for producing reduced- or nitrate/nitrite-free products [9]. The incorporation of BP into ordinary sausages has shown that the natural colorant was able to prevent lipid oxidation, enhance and maintain the products’ red color, and had minimal impact on lactic acid bacteria (important microbial genera for desired flavor compound production) during storage [10]. Aykın-Dinçer et al. [10] and Bellucci et al. [11] also observed that BP extract in sausages helps to prevent lipid oxidation and preserve sausages’ color. Additionally, both studies observed that the addition of beetroot powder enhanced the appearance/color and overall liking of the sausages compared with the control samples [10].

The Brazilian fresh sausage market was estimated at approximately USD 0.6 million in 2024 and presents a growing potential rate of 5% annually up to 2030 [12,13]. Within the context of growing consumer concerns about the toxicological aspects of chemical preservatives and their willingness to consume more natural products [9], several studies indicate that BP has shown great potential to be used as a natural colorant in foods, providing functional properties to the product without compromising sensory acceptance [9,10,11,12]. Sausages usually contain added curing salts (nitrites and nitrates) to avoid the growth of *Clostridium botulinum* and to enhance color quality. However, they are considered potential human carcinogens (Group 2A by the International Agency for Research on Cancer), justifying current consumer concern about their ingestion [9].

Although previous studies have shown that BP enhances overall liking, it is important to further explore consumer perceptions and attitudes toward novel foods, as consumer acceptance and choice extend beyond mere liking [14]. In current sensory and consumer science research, emotions have become a critical approach for explaining acceptance data, providing a holistic understanding of consumers’ affective product experiences and clarifying why liking may not always predict market success [14,15]. Among the various tools used to evaluate emotions, the 25-item EsSense25 [16] is widely applied. It is a shortened version of the original 39-item EsSense Profile [14], which was developed by statistically selecting the emotions most relevant to food experiences. EsSense25 has proven to be an important tool for discriminating among samples and for understanding consumers’ affective responses to food products [14,16].

Understanding the drivers of food purchasing is an important aspect for developing more effective marketing strategies. Among the various applications of projective techniques, one possible use is to explore consumer beliefs, feelings, and motivations related to purchasing behaviors. Projective techniques employ structured stimuli that allow consumers to externalize perceptions and emotional associations that may not emerge through direct questioning, providing a deeper understanding of underlying consumer responses [17]. Within this framework, completion tasks have been applied not only to access behavioral tendencies related to purchasing but also to estimate acceptable price ranges for products [18]. Completion tests have been used, for example, to investigate consumer drivers and willingness to pay for products such as honey, ready-to-eat salads, and frozen burgers [17,18,19,20,21].

To date, very little is known about the emotional responses and purchasing factors associated with BP in nitrite-free sausages, and consumer preference alone is not enough. Understanding emotions, motivations, and willingness to pay is critical to market success [14,21]. Therefore, the aim of this study was to evaluate the effects of different concentrations of beetroot powder in nitrite-reduced fresh sausages on consumers’ liking, emotional responses, and willingness to pay, and to identify the purchasing factors that influence these responses.

## 2. Materials and Methods

### 2.1. Ingredients

Pork leg meat and *toucinho* (subcutaneous pork fat with rind) were purchased from a local butcher (Lajeado, RS, Brazil). Salt (Sal Sul, Imbituba, SC, Brazil), dehydrated garlic and onion mix (Luar Sul, Santa Cruz do Sul, RS, Brazil), and BP (Du Loiro, Jundiaí, SP, Brazil) were purchased from a local market (Lajeado, RS, Brazil).

### 2.2. Production of Fresh Sausages

Fresh sausage production followed the procedure proposed by Bellucci et al. [11], with few modifications. Two kilograms and five hundred and fifty grams of pork meat (2.55 kg) and 1.8 kg of bacon, at ~7 °C, were ground using 12 mm disk equipment (CAF, model 22S, Rio Claro, SP, Brazil), then mixed with salt and the dehydrated seasoning mix. After a homogeneous dough was obtained, the BP was added in different amounts as shown in Supplementary Table S1. The trials proposed were (i) control, without BP; (ii) F1, with the addition of 15 g of BP (3.4 g/kg for BP amount/sausage weight); (iii) F2, with the addition of 30 g of BP (6.8 g/kg); (iv) F3, with the addition of 45 g of BP (10.2 g/kg) (Table S1). The mixtures were stuffed into natural tripe with a 28 mm diameter, and these casings were knotted at 15 cm intervals. Since the objective of the present work was to evaluate fresh sausages, no maturation period was applied to the samples. Sausages were frozen at −18 °C [11] in a domestic freezer for 15 days and thawed at 7 °C overnight before analysis.

### 2.3. Instrumental Characterization of Samples

In order to characterize the samples, moisture, water activity (Aw), color, and cooking loss were measured. All analyses were performed with cooked samples. In this context, sausages were placed onto Teflon trays and cooked at 220 °C for 20 min, then removed and left to cool to 25 °C before instrumental analysis.

Moisture and pH were evaluated following standard methods from the AOAC [22]. Water activity (Aw) was measured using a Novasina Labswift-aw (Lachen, Switzerland). Color parameters were evaluated at three points on the sausages’ surface by the CIELAB (L*, a*, and b*) parameters, using the D65 diffuse illumination of a colorimeter (Minolta Chroma CR-400). Color difference (ΔE) was measured according to Equation (1) [23].

(1) $$∆E=\sqrt{{\left(L_{control}-L_{F}\right)}^{2}+{\left(a_{control}^{*}-a_{F}^{*}\right)}^{2}{+\left(b_{control}^{*}-b_{F}^{*}\right)}^{2}}$$

Cooking loss was calculated from the difference between sausage weights before ($W_{bc}$) and after ($W_{ac}$) the cooking procedure as follows (Equation (2)):

(2) $$Cooking\;loss\left(\%\right)=\frac{W_{bc}-W_{ac}}{W_{ac}}\times 100$$

### 2.4. Consumer Test

#### 2.4.1. Recruitment

All volunteers (n = 91) were recruited in Lajeado (29°28′7″ S, 51°57′55″ W) through posters on the university campus and via social media according to the following criteria: over 18 years old, not pregnant, non-lactating, sausage consumer, and willing to participate. There was no financial incentive for participants to attend the test. In line with the principles of the Declaration of Helsinki, before participation, all volunteers read and signed the Free and Informed Consent Form. Consumers were not aware of the objective of the project (BP in the sausage formulation) to avoid biases. The experimental procedure was approved by the Ethics Committee of the State University of Rio Grande do Sul (approval certificate number 79331924.9.0000.8091).

#### 2.4.2. Sensory Analysis Procedure

Cooked sausages (Section 2.3), after oven cooking, were cooled to 60 °C at room temperature (~25 °C). For sensory analysis, sausages were served at 60 °C (temperature of consumption) [8,10]. The temperature was monitored by an infrared surface thermometer. Then, sausage slices (3.0 cm in diameter and 0.3 cm in height) were cut and kept at 60 °C until sensory analysis in an oven at 60 °C, and the surface temperature of the samples was monitored every 15 min. The maximum waiting time for samples was 20 min to avoid changes in the samples.

The sensory evaluation was conducted in a neutral, centralized testing room maintained at 20 ± 2 °C, under white lighting, free from extraneous odors, and separated from the sample preparation area. Samples from a single batch (~4.4 kg; Table S1) were presented monadically on plastic plates labeled with three-digit random codes, following a complete and balanced design to minimize order effects [24]. To avoid carryover between samples, participants were instructed to cleanse their palate with filtered water between evaluations.

Initially, volunteers tasted each sausage sample and assessed their overall liking using a 9-point hedonic scale, anchored at “dislike very much” and “like very much”. Subsequently, participants followed the EsSense25 emotion lexicon by selecting all the terms that reflected the emotions they experienced while tasting each sample. Following the emotional assessment, participants evaluated the samples using a 5-point just-about-right (JAR) scale (1 = “Not enough,” 3 = “Just about right,” 5 = “Too much”) for three specific attributes: color, bitterness, and flavor. These attributes were selected based on previous findings that color is closely related to the acceptance of sausages formulated with beetroot powder (BP) [9,11], and that polyphenols, abundant in BP, contribute to a bitter taste and earthy flavor [25,26].

Finally, within the projective approach [17], participants completed two sentence completion tasks designed to explore price perception and purchase motivation. The first sentence to be completed was stimulated by a photograph with a speech bubble showing a middle-aged man holding a sausage in front of a supermarket shelf with several cured meats, thinking: “The kilogram of an ordinary sausage is R$20.00; then this one without nitrite and nitrate and added with beetroot powder may cost R$ __________ per kilogram.” Then, a picture of a middle-aged couple in a grocery store in front of a table full of cured meats was shown, where the man says, “Have you seen this sausage without nitrite and nitrate and added with beetroot powder?” and the woman answers, “Interesting! Let’s buy it because ____________.” Photographs were built by artificial intelligence using Canva software as proposed by De Souza et al. [20] and followed recommendations from Vidal et al. [19] to present in the figures: (i) adults; (ii) with no fancy clothes; (iii) who were happy to be in the situation; (iv) using ordinary terms and words to make the characters relatable to the respondents.

### 2.5. Data Analysis

Analysis of variance (ANOVA), Cochran’s Q test, correspondence analysis (CA), and penalty analysis (PA) were performed using XLSTAT software (Addinsoft, New York, NY, USA, version 2022.3.1—https://www.xlstat.com/, accessed on 21 October 2025), while Shapiro–Wilk’s, Hartley’s maximum F, and Generalized Linear Model (GLM) tests were performed using R software version 4.0.5 (31 March 2021) (www.r-project.org/). The alpha risk was set at 5%.

#### 2.5.1. Instrumental Data

Data (means of triplicates from two independent experiments) from moisture, Aw, color parameters, and cooking loss were compared by one-way ANOVA followed by Tukey’s Honest Significant Difference (HSD) post hoc test.

#### 2.5.2. Overall Liking and Emotional Data

To evaluate statistical differences in consumer liking scores, means were analyzed by two-way ANOVA followed by Tukey’s HSD post hoc test, considering *p* ≤ 0.05 as significant. In ANOVA, samples were considered a fixed effect and consumers a random effect. The normality of the data and the homogeneity of variances were evaluated by the Shapiro–Wilk’s and Hartley’s maximum F tests, respectively, and were considered satisfactory when *p* > 0.05.

Frequencies of mention for each emotion term were determined by counting the number of consumers who checked each term to describe each sample, and the nonparametric Cochran’s Q test was applied to detect differences in consumers’ perception of the evaluated samples. Differences were considered statistically significant when *p* < 0.05. To obtain the perceived sensory maps of the samples, either for sensory attributes or emotions, correspondence analysis (CA), based on chi-squared distances and the test of independence between rows and columns, was applied to the frequency table containing the number of consumers who checked each term from the CATA tasks (emotion terms) to describe each sausage. For CA, sensory and emotional items were considered only when they showed statistical differences among samples (*p* < 0.05). The statistical relationship between emotions and products was evaluated by the chi-square test and was considered significant when *p* < 0.05.

#### 2.5.3. JAR Data

Penalty analysis (PA), following Lawless and Heymann [27], was applied to the results obtained from the JAR analysis. In addition, the frequencies of ratings 1 and 2 were counted and considered “too little”; 3, “ideal”; and 4 and 5, “too much.”

#### 2.5.4. Completion Tasks

All sentences initially underwent the lemmatization procedure (the correction of typing/spelling mistakes, the deletion of connectors and auxiliary terms, the standardization of verbs in the infinitive, nouns in the singular, and adjectives in the masculine singular form, and regrouping those with their synonyms). The regrouping of answers was cautiously performed to avoid the overinterpretation or overgrouping of words by using the triangulation process, in which three independent researchers separately analyzed word discrepancies, and converging ideas were discussed and resolved by the authors. Responses were then categorized based on the previous literature, and convergences were reached through the triangulation process again [20,28,29].

#### 2.5.5. Effect of Driver of Purchasing on Liking and Emotions

Consumers were split into two clusters of purchasing drivers based on the results from Section 2.4.2. To evaluate the differences in consumer liking, mean liking scores were analyzed by two-way ANOVA (sample and cluster as fixed factors) to ascertain any significant differences or interactions between clusters and samples. Since emotions were assessed by binary nonparametric data, the effects of sample, cluster, and interaction (sample*cluster) on responses were evaluated by the Generalized Linear Model (GLM) [30]. The GLM default function = glm, family = binomial, and link = logit [30] was used to model the citation proportions of the two factors (sample and cluster) with interactions for each attribute. An Analysis of Deviance and associated chi-square tests were used to determine the statistical significance of the sample, group, and respective interactions [31]. Post hoc tests were subsequently conducted using Tukey’s HSD via the emmeans package for pairwise comparisons. When the interaction was not significant (*p* > 0.05), it was removed from the model for post hoc tests. For terms without significant interaction effects but with significant main effects, the GLM was re-run with only the main effects, followed by Tukey’s HSD post hoc.

In addition, to evaluate the relationship between consumers’ sociodemographic profiles and the results from purchasing drivers, an independent chi-square test was performed, followed by Fisher’s exact test per cell, to identify relationships between the sociodemographic profile and the purchasing categories. In this analysis, the frequency of consumption was considered frequent when consumers claimed to consume sausages “several times a month, but not every week” or more frequently, and occasional when they claimed to consume sausages “once a month” or less frequently.

## 3. Results and Discussion

### 3.1. Characterization of Sausages

The instrumental characterization of sausages used in the consumer tests is shown in Table 1. The results show that moisture tended to decrease with the addition of BP into the samples’ formulation, as well as Aw. The highest values of moisture were observed in the control and F1, which did not differ from each other (*p* > 0.05), and the lowest values in F2 and F3. Aw did not differ among the control, F1, and F2 (*p* > 0.05), and F3 presented the lowest values, which did not differ from F2. These phenomena are related to the high water-holding capacity of BP [32]. The pH values indicated that the addition of BP resulted in higher acidity of the samples compared with the control (*p* < 0.05), and the different amounts of BP did not affect the sausages’ pH (*p* > 0.05). L* values are related to the samples’ lightness [23], and the results showed that samples with BP became darker. a* values are related to the reddish color [23], and the addition of BP resulted in redder samples, possibly due to the higher amount of betalains. Positive b* values are related to the yellowish color of samples [23], and results indicated no statistical differences among samples (*p* > 0.05). ΔE values indicated differences higher than 2.3, which is considered the threshold for visually noticeable differences [33], indicating that the addition of BP caused visible changes to the naked eye. Regarding cooking loss, samples with BP presented lower values (*p* < 0.05) compared with the control, and F2 and F3 presented the lowest parameter values (*p* > 0.05), possibly due to the water-holding capacity of BP [32].

### 3.2. Consumers’ Sampling

Consumers’ profiles involved in the present study are presented in Supplementary Table S2. The sample was composed of 63.7% (n = 58) women, with an average age of 28.5 ± 10.2 years. The majority of consumers had completed high school or higher education (94.5%, n = 86), and 59.3% (n = 54) earned between one and three minimum salaries. Regarding sausage consumption, 50.6% (n = 46) reported eating sausages several times a month or more frequently.

### 3.3. General Results

**Figure 1:**
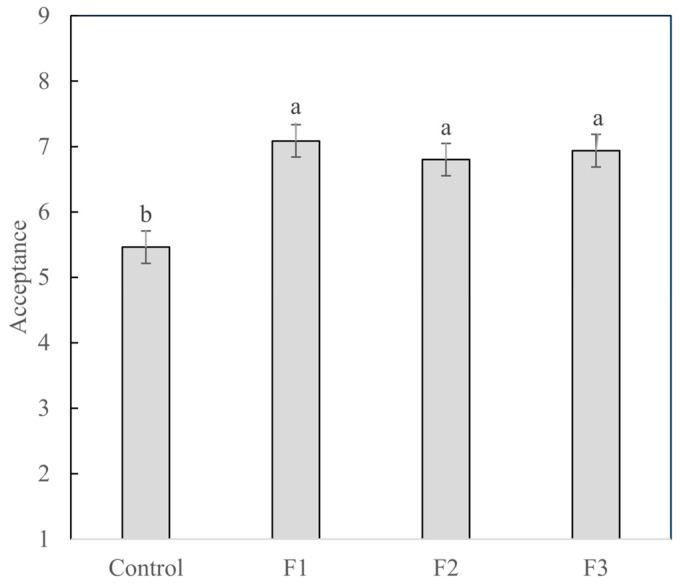
Average liking scores for fresh sausages with different amounts of added BP. ^a,b^ different letters indicate statistical differences among samples according to Tukey’s test.

The results of overall liking are shown in Figure 1. Fresh sausages with added BP (F1, F2, and F3) showed average overall liking scores between 7.2 and 6.8, which were significantly higher (*p* < 0.05) than the scores for samples without the colorant.

Bellucci et al. [11] and Aykın-Dinçer et al. [10] also observed that the addition of BP increases the overall and color acceptance of sausages compared with control samples without any colorant. Acceptance could be impacted by undesirable flavors (such as a bitter taste and earthy flavor) present in BP [25,26], but the results herein corroborate the literature indicating that BP does not bring this limitation to practical applications. The results are in line with the findings of Aykın-Dinçer et al. [10], who observed that the increase in the acceptance of sausages with BP was directly related to the products’ color, possibly due to the fact that beetroot provided a more natural color to the sausages [9]. It is important to point out that Sucu and Turp [5] also observed that, at the very beginning of their shelf life, sausages with BP presented higher acceptance, but over storage time, there was no significant difference between samples. Therefore, further studies are important to advance the understanding of BP addition as a substitute in reduced/low-nitrite/nitrate foodstuffs.

### 3.4. Emotions Evoked

**Figure 2:**
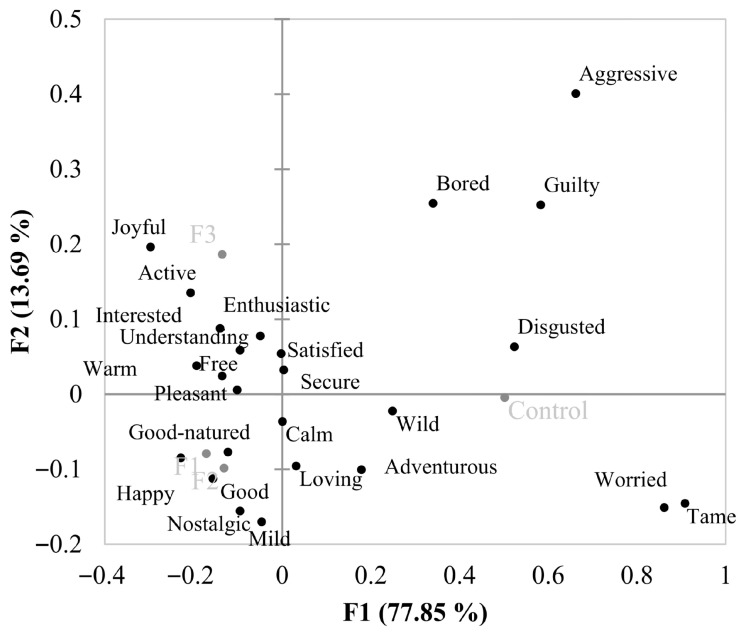
Biplot of CA emotions evoked for fresh sausages with added BP. Emotions are expressed in black and samples in gray.

The results from counting the frequency of checked emotion terms when consumers tested each sample are presented in Table 2, and Figure 2 shows the affective map obtained from these data, performed using the CA methodology. “Joyful” and “Pleasant”, emotions with positive valence [14], were cited with higher frequency when consumers tasted samples with BP (Table 2). On the other hand, “Worried” and “Disgusted”, negative emotions, presented higher citation frequencies for the control sample. “Tame,” which has no clear classification, showed a higher citation frequency for the control. These results are in line with the most liked and disliked sausages evaluated, where higher liking scores were associated with positive emotions and lower scores with negative ones. “Active”, “Good-natured”, “Good”, and “Happy”, all positive emotions, as well as “Mild”, “Aggressive”, and “Understanding”, emotions with no clear classification, presented significant differences in citation frequency, but neither control nor BP samples stood out for a higher citation (Table 2).

The results show that samples with added BP were placed on the opposite side of the biplot graph (Figure 2) from the control samples. F3 evoked mainly “Joyful”, “Warm”, “Free”, “Secure”, “Active”, “Pleasant”, “Enthusiastic”, and “Interested”, which present positive valence [14]. F2 and F3 evoked “Happy”, “Nostalgic”, “Good”, and “Good-natured”, which are also positive emotions. Rocha et al. [34] also observed that frankfurters with health claims evoke positive emotions. “Mild” was located close to F1 and F2, whereas “Understanding” was located close to F3. Both emotions are considered to have no clear classification, with their connotation depending on the product and context [15,16].

Control samples evoked “Worried”, which is a negative emotion, and “Wild” and “Tame”, which do not have a clear classification [14], but in the present context, they have a negative connotation. The control sample also evoked “Adventurous”, which is a positive emotion. Eating is considered a positive experience for most people, and positive emotions may be evoked even when consumers show low liking for a product. Additionally, control samples showed an overall liking score of 5.4, indicating that the sausages were rated between “neither liked nor disliked” and “slightly liked”, which helps to explain the association of a positive emotion positioned closer to the most disliked sample. The results herein are in line with the findings of Rocha et al. [34,35], who observed that “Calm”, “Guilty”, “Glad”, and “Nostalgic” were more frequently applied to ordinary frankfurters when compared with frankfurters with a healthier appeal.

### 3.5. JAR

The results of the penalty analysis based on the JAR test are presented in Table 3. When color, flavor, and bitterness were considered “too little” in the samples tested, overall liking was significantly and negatively affected (*p* < 0.05) by 1.4, 1.8, and 1.1 points, respectively. When color, flavor, or bitterness were considered “too much,” no significant (*p* > 0.05) effect was observed on liking. Beetroots are phenolic-rich plants, which may bring a bitter taste and astringent flavors to foods [26], and also contain critical amounts of geosmin, which imparts earthy flavors [27]. The negative effect of a low amount of BP in the formulations may be related to the samples’ lower color intensity, which has been correlated with sausage liking when BP was used as a natural colorant [5]; this perception might have carried over to flavor and bitterness scores. The neutral effect of a high amount of BP in the formulation provides important results for the food industry to explore the natural colorant in cured meats.

**Figure 3:**
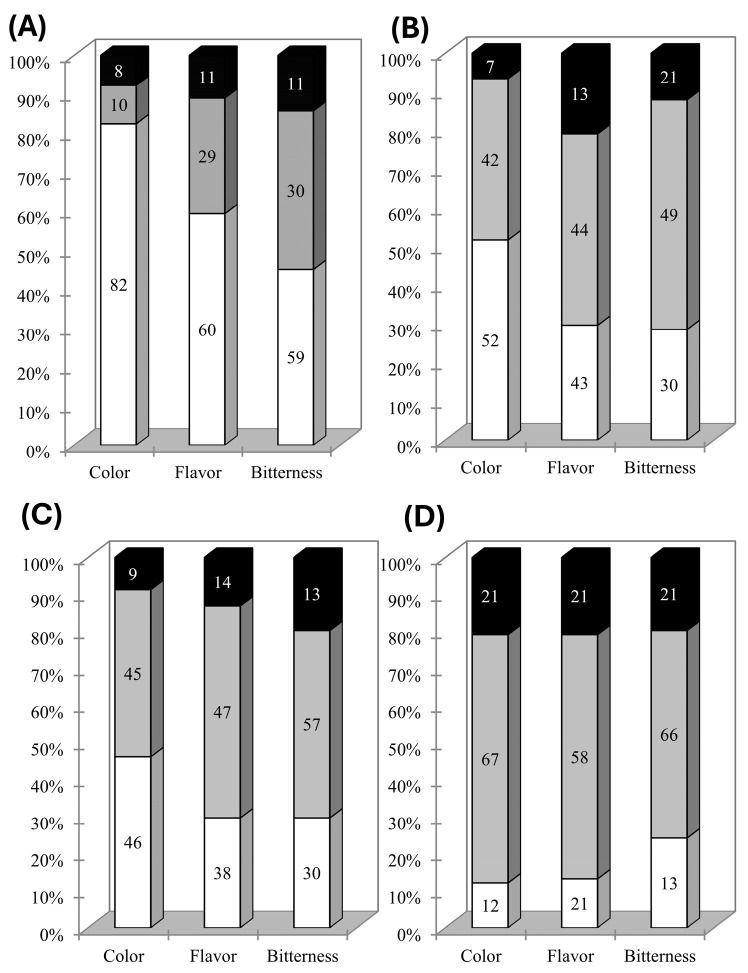
Frequency of citation of the collapsed levels (too little, white; ideal, gray; too much, black) for control (**A**), F1 (**B**), F2 (**C**), and F3 (**D**) sausage samples.

Figure 3 shows the proportion of citations for the JAR levels of each sample. Figure 3A shows that sausages without any colorant (control samples) were considered to present mainly “too little” color, flavor, and bitterness. The addition of BP into the samples’ formulation led to an increase in the ideal proportion of citations for the three attributes evaluated, indicating a positive effect of BP on sausage production. The increase in the proportion of the ideal level from the control to the BP-added samples (Figure 3) is related to the instrumental color analysis showing the increase in a* and L* values of the samples (Table 1). Additionally, the proportion of “too much” also increased, but the penalty analysis (Table 2) indicated that this level does not impact the liking of the samples. BP brings a bright red-violet color to foods, similar to the red-brown color that the Maillard reaction imparts to cooked foods (in line with the a* and L* values found in Table 1), which may help explain the results in the present work. Moreover, color has been shown to be significantly related to liking when sausages are formulated with BP [5], whereas bitterness is usually considered a driver of disliking in foodstuffs [26]. However, the results indicate that the addition of BP in the amount used herein does not impart any negative bitterness to the sausages.

Ávila et al. [36] indicated that a proportion of citations above 70% means that the attribute exerts a strong influence on the overall impression, whereas below 20% the attribute should not be considered significant for the overall impression. Thus, considering the negative impact on liking when color, flavor, and bitterness were below the ideal level (Table 2), the results from the JAR test indicate that the F3 formulation was the most suitable to be considered for further studies, which is in line with the results in Figure 1.

### 3.6. Sentence Completion

The results from the projective technique used to access purchasing drivers are presented in Table 4. Health was the most cited reason, cited by 51.6% (n = 47) of participants, followed by natural/without chemicals (24.2%, n = 22), hedonic aspects (9.9%, n = 9), and interesting/different (7.7%, n = 7). Rocha et al. [34] showed that health is the most frequent citation when consumers think about frankfurters with natural antioxidants, with sodium or fat reduction and with omega-3. Respondents that did not answer the completion question composed 6.6% (n = 6) of the sample.

Consumers have demonstrated an awareness of a potential link between meat consumption and cancer [37], and they generally exhibit a favorable intention to purchase products with reduced nitrate/nitrite levels and with incorporated natural compounds [38]. This is in line with the results of the present work. Thus, exploring the healthiness of sausages with added BP and low/reduced-nitrite and nitrate claims are important strategies to enhance these products’ consumption, since consumers tend to prefer claims that refer to a reduction in the risk of common lifestyle-related diseases rather than claims that promote direct health benefits [39]. Regarding the “Interesting/Different” category, other works have related these terms to novel products [28,29]. De Barcellos et al. [40] showed that Brazilian consumers were not the most inclined to adopt innovations, but they were not afraid of new foods either, which may help to explain the driver of purchasing and liking results described in Section 3.3.

In the sentence completion task for perceived price (where the average price of one kilogram of ordinary sausage was set at BRL 20.00 (~USD 3.42)), consumers indicated that they would pay BRL 28.00 (~USD 4.79) (40% more compared with the ordinary sausage—BRL 20.00) for a product without nitrite and nitrate and with added BP. The literature indicates that higher prices were associated with frankfurters containing natural antioxidants and those enriched with omega-3 [34]. Also, consumers have claimed to be willing to pay slightly more for innovative cured meats perceived as healthier [37,41], whereas in the present work, consumers indicated that they would pay 40% more. However, the results show that 62.6% (n = 57) would pay more for the healthier version, while 37.4% (n = 34) reported that they would not pay more (equal to or less than the regular price). Thus, the results show that food industries may be missing opportunities to innovate further in meat products, especially in reduced/low-nitrite/nitrate products and/or those with natural dyes, although consumer clustering based on price remains important.

### 3.7. Influence of Purchasing Drivers on Liking and Emotions

Consumer behavior and attitudes toward food involve complex decision-making processes, and their segmentation is essential nowadays for the proper evaluation of how consumers react to foods, enabling the food industry to better target specific demographic groups and refine logistics and marketing strategies [42,43]. In the present work, consumers were split into two purchasing groups: those who claimed healthiness as a purchasing driver of the samples and those who claimed other motivations. This segmentation resulted in a sample distribution close to 50% (the health-oriented segment was composed of 51.6% (n = 47) and other drivers of 48.4% (n = 44)). Although sample sizes in the two segments were not as large as recommended for consumer tests, they exceeded the minimum of 40 for stable estimates [44], and these sample sizes have been used before to explore consumers’ behavior in food acceptance studies [45,46].

The results of the statistical relationship of consumers’ profile and both clusters (Supplementary Table S3) showed that purchasing driver was not related to gender (c^2^_observed_ = 0.081; c^2^_critical_ = 3.841; *p* = 0.776), monthly income (c^2^_observed_ = 3.001; c^2^_critical_ = 9.488; *p* = 0.063), and the frequency of sausage consumption (c^2^_observed_ = 0.011; c^2^_critical_ = 3.841; *p* = 0.916). However, educational level was statistically correlated with clusters (c^2^_observed_ = 9.525; c^2^_critical_ = 7.815; *p* = 0.023), and the results showed that consumers with postgraduate education were associated with the health-oriented cluster, whereas those who had completed high school belonged to the “Other purchasing driver” group.

**Figure 4:**
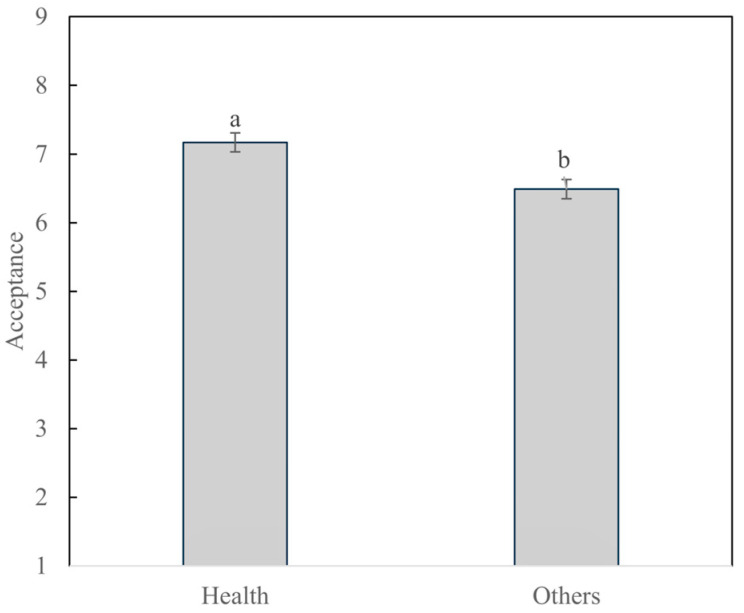
Average acceptance scores of BP sausage samples for the different cluster of drivers of purchasing. ^a,b^ Different letters indicate statistical differences between clusters according to Tukey’s test.

The results from the two-way ANOVA showed, when analyzing overall liking, significant differences (*p* < 0.05) between clusters (*p* = 0.001) and among samples (*p* < 0.0001), but the interaction between them (cluster*sample) was not significant (*p* = 0.822). Since the interactions were not significant, the results from the samples remained the same as observed in Section 3.1. Figure 4 shows the differences between clusters. Health-oriented consumers, whose main driver was purchasing sausages without nitrite/nitrate and with added BP, presented higher (*p* < 0.05) overall liking (Figure 4). Supplementary Table S4 shows the contingency table of both segments and whether they claimed they would pay a premium price for a low-nitrite/nitrate BP sausages or not (those who claimed they would pay less or equal were grouped together). The results from the χ^2^ test (χ^2^_observed_ = 0.007; χ^2^_critical_ = 3.841; *p* = 0.935) indicate no relationship between the cluster and willingness to pay more for the novel meat product proposed.

Consumers have indicated that they are unwilling to compromise on the sensory characteristics of cured meat [47]. However, they also tend to perceive cured meat as healthier when it contains high-quality meat as a raw material, is formulated with reduced salt or nitrite levels, incorporates natural antioxidants and healthier ingredients, or possesses modified fat profiles [13,36]. In this context, Sabbe et al. [48] observed that health-oriented consumers were more likely to compromise on taste for a potential health benefit. However, there is a share of consumers who consider frankfurters labeled as “no nitrite” to be “not appealing” and having “flavor defects”, and they rated those products with the lowest purchase intention [35]. Additionally, the literature suggests that consumers have limited awareness regarding both the presence of nitrates/nitrites in cured meats and their potential effects [2], which may help explain the results observed in the present work.

An evaluation of the influence of the purchasing driver on the emotions evoked is shown in Table 5. Only “Active” and “Worried” presented significant differences in the interaction of cluster*sample (*p* < 0.05) in the deviance analysis. However, based on Tukey’s test following the GLM analysis, no significant pairwise differences were found across samples or clusters for either emotion. This behavior is not uncommon when the frequency of term citation is low [49]. “Active”, “Calm”, “Enthusiastic”, “Free”, “Loving”, “Secure”, and “Disgusted” presented significant differences between clusters, whereas “Joyful”, “Understanding”, “Aggressive”, “Disgusted”, “Good”, “Guilty”, “Happy”, “Tame”, and “Worried” presented significant differences among samples.

**Figure 5:**
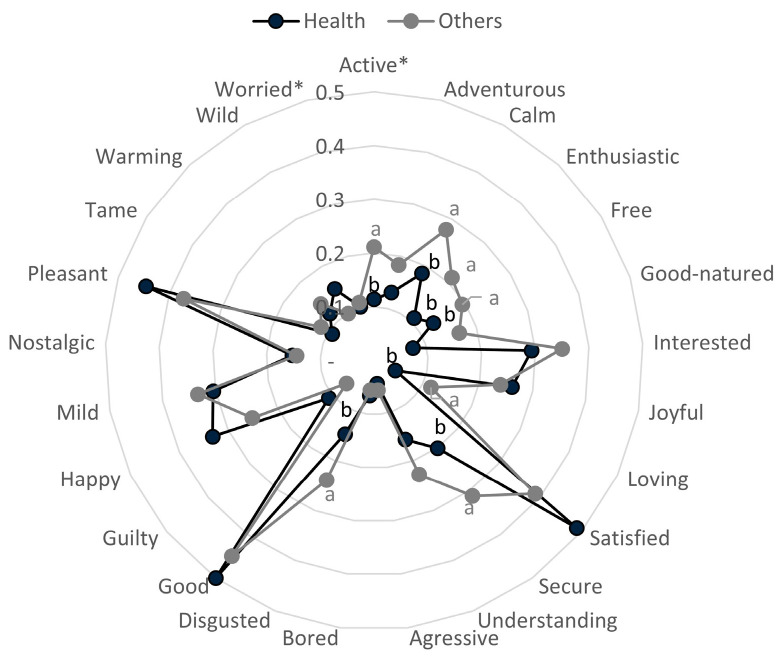
Portion of citation of emotion items for the different clusters of drivers of purchasing. ^a,b^ Different letters indicate statistical differences among clusters according to the GLM test. * presented significant differences in the interaction of cluster sample (*p* < 0.05) in the deviance analysis.

Figure 5 shows the differences in emotions evoked by the different clusters. Health-oriented consumers presented a lower frequency of citation for all emotions with significant differences between clusters. This is surprising, since the health-oriented cluster presented higher liking scores for the samples. The results may be explained by the fact that individuals in a positive mood tend to prioritize long-term goals, such as health, leading them to choose healthier foods. However, this choice may not provide the same immediate satisfaction that indulgent foods do, resulting in a lower evocation of positive emotions despite the high acceptance of the product [50]. De Brito et al. [51] observed that Brazilian health-oriented consumers preferred less indulgent products than consumers from other clusters. Consumers in the “Other” purchasing driver group presented hedonic feelings (9.9%), interest in trying the sausage (7.7%), or indifference (6.6%, who did not answer the sentence completion task) as the main reasons to buy a sausage without nitrite/nitrate and with added BP, indicating that their consumption could be related to indulgent factors, leading to a higher arousal of emotions evoked.

Table 6 shows the penalty analysis of the JAR tests for both clusters evaluated. In line with the general results (Table 2), for both clusters, when color, flavor, and bitterness were considered “not enough”, there was a significant negative effect on liking scores (*p* < 0.05). When color intensity was in excess, liking was not affected (*p* > 0.05) for health-oriented consumers, whereas for other consumers, the samples were not perceived to present this behavior. However, when flavor and a bitter taste were considered to be in excess, liking scores were significantly reduced only for health-oriented consumers (*p* < 0.05) and were not affected for other consumers (*p* > 0.05). Thus, health-oriented consumers proved to be more susceptible to the addition of a high amount of BP, indicating that they are unlikely to compromise liking when high bitterness is perceived. Although the literature suggests otherwise—i.e., that health-oriented consumers tend to be less critical of flavor drawbacks—these consumers still preferred the best-tasting product [48]. This behavior can be seen in Table 5, where health-oriented consumers who perceived bitterness in excess still gave an average liking score of 6.8, indicating that they still partially liked the products.

## 4. Limitations

A total of 91 consumers took part in this study, and it would be prudent to confirm the stability of the current results using a larger sample size (e.g., over 112 individuals [52]), particularly among consumers with different purchasing drivers to ensure a more representative distribution. Also, the sampling does not represent the Brazilian profile and is limited to the Lajeado (RS, Brazil) region.

Sausages in this study were prepared using a single recipe protocol. Variations in ingredients, their proportions, and modifications to the production process could influence the results. Among further sausage recipes to be evaluated, the addition of a control sample containing chemical nitrite/nitrate would help compare results to BP-added sausages. Additionally, results are restricted to the conditions established in the methodology section (such as the emotion list, short storage time, and period context). Thus, further research should explore a broader selection of ingredients and social/cultural contexts to better understand their interactions with BP.

## 5. Conclusions

The results of the present work show that the incorporation of BP is a valid strategy for the food industry, since it significantly enhances overall liking and evokes positive emotions. The addition of BP did not introduce undesirable bitterness or earthy flavors, which are commonly associated with phenolic-rich plants like beetroot, but improved the perceived balance of color, flavor, and bitterness in the sausages evaluated. Consumers claimed to be willing to pay a higher price for a sausage with BP and without nitrites and nitrates, and the main purchasing drivers were the healthiness and naturalness of this kind of product. This suggests that BP is a viable natural alternative to synthetic colorants, capable of improving the sensory properties of sausages.

The results herein also showed that health-oriented consumers presented higher overall liking for sausages with BP, underscoring the importance of health claims in influencing consumer behavior. However, these consumers were more sensitive to excessive bitterness, indicating a nuanced balance between health benefits and sensory liking. Additionally, health-oriented consumers evoked fewer positive emotions than other consumers.

In conclusion, the results provide strategic and unique information about consumers for the food industry, aiming to support the development of innovative food products that not only meet sensory expectations but also align with health-conscious consumer preferences. The results highlight the need for food industries to carefully calibrate the amount of BP in formulations to optimize both health appeal and sensory satisfaction.

## Figures and Tables

**Table 1:** Physicochemical characteristics of fresh sausage samples.

	Control	F1	F2	F3
Moisture (%)	93.86 ± 1.53 ^a^	90.50 ± 1.33 ^a^	88.18 ± 2.24 ^b^	87.96 ± 0.87 ^b^
Aw	0.992 ± 0.002 ^a^	0.992 ± 0.001 ^a^	0.991 ± 0.001 ^ab^	0.988 ± 0.003 ^b^
pH	5.66 ± 0.03 ^a^	5.46 ± 0.04 ^b^	5.41 ± 0.04 ^b^	5.39 ± 0.05 ^b^
L*	54.72 ± 0.23 ^a^	50.67 ± 3.02 ^a^	43.84 ± 1.05 ^b^	40.23 ± 2.45 ^b^
a*	1.77 ± 0.26 ^c^	3.47 ± 0.62 ^b^	4.59 ± 0.40 ^ab^	6.12 ± 0.15 ^a^
b*	0.55 ± 0.11 ^a^	0.71 ± 0.07 ^a^	0.67 ± 0.05 ^a^	0.53 ± 0.05 ^a^
ΔE		4.40	11.24	15.13
Cooking loss (%)	15.29 ± 0.70 ^a^	13.07 ± 0.41 ^b^	10.19 ± 1.21 ^c^	8.23 ± 0.75 ^c^

^a,b,c^ different letters indicate statistical differences among samples based on Tukey’s test at 5% significance.

**Table 2:** Portion of citation of emotions by consumers for fresh sausages with different amounts of added BP. Control: without BP; F1: 3.5 g/kg BP; F2: 7.0 g/kg BP; F3: 7.5 g/kg BP.

Emotion	Control	F1	F2	F3	*p*-Values
Active	0.0879 ^b^	0.1538 ^ab^	0.1868 ^ab^	0.2198 ^a^	0.0140
Adventurous	0.1868	0.1319	0.1868	0.1209	0.3102
Calm	0.2088	0.2527	0.2418	0.2198	0.8358
Enthusiastic	0.1429	0.1648	0.1538	0.1758	0.8907
Free	0.1209	0.1868	0.1538	0.1868	0.2886
Good-natured	0.0879 ^b^	0.1978 ^a^	0.0879 ^b^	0.1099 ^ab^	0.0087
Interested	0.2637	0.3516	0.2967	0.3736	0.2115
Joyful	0.0989 ^a^	0.2637 ^a^	0.2637 ^a^	0.3736 ^a^	0.0000
Loving	0.0769	0.0989	0.0769	0.0659	0.6912
Satisfied	0.3956	0.4066	0.4835	0.4725	0.4098
Secure	0.1868	0.2857	0.2747	0.2747	0.2302
Understanding	0.1319 ^b^	0.2747 ^a^	0.1319 ^b^	0.2308 ^ab^	0.0039
Aggressive	0.0989 ^a^	0.0220 ^b^	0.0110 ^b^	0.0659 ^ab^	0.0064
Bored	0.0879	0.0330	0.0440	0.0769	0.2530
Disgusted	0.3407 ^a^	0.1209 ^b^	0.1429 ^b^	0.1648 ^b^	0.0002
Good	0.3077 ^c^	0.6154 ^a^	0.5495 ^ab^	0.4286 ^bc^	0.0000
Guilty	0.1648 ^a^	0.0549 ^b^	0.0330 ^b^	0.0989 ^ab^	0.0004
Happy	0.1538 ^b^	0.3956 ^a^	0.3297 ^a^	0.2857 ^ab^	0.0001
Mild	0.2637	0.3736	0.3956	0.2418	0.0239
Nostalgic	0.1099	0.1648	0.1978	0.1209	0.1260
Pleasant	0.2747 ^b^	0.4396 ^a^	0.4615 ^a^	0.4615 ^a^	0.0049
Tame	0.2527 ^a^	0.0549 ^b^	0.0769 ^b^	0.0330 ^b^	0.0000
Warm	0.0769	0.1648	0.1319	0.1538	0.1193
Wild	0.1648	0.0879	0.1429	0.1099	0.1949
Worried	0.2527 ^a^	0.0879 ^b^	0.0549 ^b^	0.0330 ^b^	0.0000

^a,b,c^ Different superscript letters indicate significant differences (*p* < 0.05) between sausages according to Cochran’s *Q* test.

**Table 3:** Effect of different sausages’ attributes based on JAR tests and mean impact on liking from penalty-lift analysis. * significant mean impact based on penalty-lift analysis (*p* < 0.05).

Attribute	Level	Mean	Mean Drop	*p*-Value
Color	Too little	6.1	1.4	<0.0001 *
	JAR	7.6		
	Too much	7.1	0.5	0.266
Flavor	Too little	5.8	1.8	<0.0001 *
	JAR	7.5		
	Too much	6.9	0.6	0.058
Bitterness	Too little	6.2	1.1	<0.0001 *
	JAR	7.3		
	Too much	6.6	0.7	0.061

Too little: rates 1 and 2 on JAR scale; JAR: rate 3; too much: rates 4 and 5 on JAR scale.

**Table 4:** Categories for the answers of the sentence completion, with number and frequency of citations.

Category	Example	n	f (%)
Health	“It is healthier”; “The product is healthier”	47	51.6
Natural/without chemicals	“It is more natural”; “there is no chemical preservatives”	22	24.2
Hedonism	“Looks good”; “looks delicious”; “it is tastier”	9	9.9
Interesting/different	“It is interesting”; “It is different”	7	7.7
Did not answer		6	6.6

**Table 5:** Deviance and associated Analysis of Deviance *p*-values regarding cluster, sample, and their interaction effects on emotions evaluated. * values indicate statistical significance (*p* < 0.05).

Emotions	Cluster		Samples		Cluster*Samples	
	Deviance	*p*-Value	Deviance	*p*-Value	Deviance	*p*-Value
Active	6.3715	0.0116 *	6.8956	0.0753	7.8487	0.0492 *
Adventurous	1.9337	0.1644	2.5939	0.4586	1.5246	0.6766
Calm	4.4522	0.0348 *	0.6273	0.8901	5.3298	0.1492
Enthusiastic	7.2186	0.0072 *	0.4190	0.9363	3.1256	0.3727
Free	2.7569	0.0983	2.0774	0.5561	3.1039	0.3759
Good-natured	7.1606	0.0075 *	6.6401	0.0842	2.9843	0.3940
Interested	1.3336	0.2482	3.1920	0.3630	3.0084	0.3903
Joyful	0.2346	0.6281	20.3901	0.0001 *	3.8927	0.2732
Loving	6.8595	0.0088 *	0.7087	0.8711	0.9936	0.8028
Satisfied	3.7188	0.0538	2.2561	0.5210	6.3770	0.0946
Secure	5.8196	0.0159 *	3.2387	0.3563	1.6466	0.6487
Understanding	2.8946	0.0888	9.2214	0.0265 *	1.0644	0.7854
Aggressive	0.2828	0.5949	10.1603	0.0173 *	1.2795	0.7341
Bored	0.1498	0.6987	3.3871	0.3357	1.9565	0.5815
Disgusted	5.0073	0.0252 *	16.7026	0.0008 *	5.1507	0.1619
Good	0.9125	0.3394	20.6233	0.0001 *	0.8181	0.8551
Guilty	2.0256	0.1547	11.4815	0.0092 *	6.1388	0.1050
Happy	2.9387	0.0864	14.7117	0.0021 *	1.5322	0.6749
Mild	0.3521	0.5529	7.5445	0.0564	0.9481	0.8138
Nostalgic	0.0430	0.8357	3.5415	0.3154	2.9486	0.3996
Pleasant	2.0317	0.1541	9.5850	0.0224 *	1.8315	0.60811
Tame	0.5742	0.4486	26.3102	<0.0001 *	1.6869	0.6399
Warm	0.4923	0.4829	3.9460	0.2674	0.5739	0.9024
Wild	2.2606	0.1327	2.9400	0.4010	2.5048	0.4744
Worried	0.0586	0.8087	25.7125	<0.0001	8.1436	0.0431

**Table 6:** Effect of different sausages’ attributes based on JAR tests and mean impact on liking from penalty-lift analysis for both purchasing drivers’ clusters evaluated. * significant mean impact based on penalty-lift analysis (*p* < 0.05).

		Health-Oriented			Others		
Attribute	Level	Mean	Mean Drop	*p*-Value	Mean	Mean Drop	*p*-Value
Color	Too little	6.6	1.0	0.000 *	5.8	1.8	<0.0001 *
	JAR	7.6			7.5		
	Too much	7.5	0.2	0.870	5.7	1.9	n/a
Flavor	Too little	6.0	1.8	<0.0001 *	5.6	1.6	<0.0001 *
	JAR	7.8			7.2		
	Too much	7.0	0.8	<0.0001 *	6.6	0.6	0.503
Bitterness	Too little	6.5	1.1	<0.0001 *	6.0	0.9	0.049 *
	JAR	7.6			6.9		
	Too much	6.8	0.9	<0.0001 *	6.4	0.4	0.669

Too little: rates 1 and 2 on JAR scale; JAR: rate 3; Too much: rates 4 and 5 on JAR scale. n/a: significance not calculated if citation frequency was <10%.

## Data Availability

The raw data supporting the conclusions of this article will be made available by the authors on request.

## Supplementary Materials

The following supporting information can be downloaded at: https://www.mdpi.com/article/10.3390/foods14213715/s1, Table S1: Sausages’ formulation; Table S2: Profile of consumers involved in sensory analysis (n = 91); Table S3: Contingency tables and statistical relation of purchasing driver and consumers’ sociodemographic profile; Table S4: Contingency table of purchasing driver and whether people would pay a premium price for a nitrite/nitrate-free sausage with added BP.
