# Supplementary material for: Beetroot Powder as Natural Colorant in Fresh Pork Sausages: Impacts on Consumer Liking, Emotional Responses, and Identification of Purchasing Drivers
Source: Foods. 2025 Oct 30;14(21):3715. doi: 10.3390/foods14213715 (PMC12607346; doi:10.3390/foods14213715)
Supplement: Supplementary file 1 [file foods-14-03715-s001.zip › foods-3882938-supplementary.pdf]

Supplementary Material

Table S1. Sausages' formulation.

Ingredients	Control	F1	F2	F3
Pork meat	2.55 kg	2.55 kg	2.55 kg	2.55 kg
Bacon	1.8 kg	1.8 kg	1.8 kg	1.8 kg
Salt	60 g	60 g	60 g	60 g
Dehydrated garlic	3.75 g	3.75 g	3.75 g	3.75 g
Beetroot powder	0 g	15g (3.4g/kg)	30g (6.7g/kg)	45g (10.1g/kg)

Table S2. Profile of consumers involved in sensory analysis (N=91).

	n	f (%)
**Gender**		
Male	32	35.2
Female	58	63.7
Did not respond	1	1.1
**Education**		
Completed fundamental school	5	5.5
Completed high school	46	50.5
Completed college	23	25.3
Postgraduate	17	18.7
**Age**		
Mean	28.5±10.2 years old	
Maximum	65 years old	
Minimum	18 years old	
**Monthly income**†‡		
Less than 1 minimum salary	7	7.7
Between 1 and 3 minimum salaries	54	59.3
Between 3 and 5 minimum salaries	11	12.1
Between 5 and 7 minimum salaries	8	8.8
More than 7 minimum salaries	9	9.9
Did not respond	2	2.2
**Frequency of consumption**		
Several times a week, but not every day	5	5.5
Once a week	18	19.8
Several times a month but not every week	23	25.3
Once a month	13	14.3
Several times a year, but not every month	18	19.8
Once or twice a year	14	15.4

†USD 1.00 ~ BRL 5.84 as of February 2nd 2025.

‡ minimum salary in Brazil in 2024 was BRL 1.412,00 monthly .

Table S3. Contingency tables and statistical relation of purchasing driver and consumers' sociodemographic profile.

	Health	Other	*p*-value
**Gender**			
Female	29	29	0.776
Male	17	15	
**Educational level**			
Completed fundamental school	3	2	0.023*
Completed high school	18*	28*	
Completed college	11	12	
Postgraduate	14*	3*	
**Monthly income**			
Less than 1 minimum salary	3	4	0.063
Between 1 and 3 minimum salaries	24	30	
Between 3 and 5 minimum salaries	6	5	
Between 5 and 7 minimum salaries	6	2	
More than 7 minimum salaries	5	4	
**Frequency of sausage consumption**			
Frequent	23	23	0.916
Occasional	23	22	

*$p<0.05$, by chi-squared test per cell and Fisher's exact test.

Table S4. Contingency table of purchasing driver and whether people would pay a premium price for a nitrite/nitrate-free sausage with added BP.

	Would pay more	Would pay less or equal
Health	29	17
Others	28	17
